# Integrated transcriptomic and metabolomic analysis unveils heat-tolerance-associated flavonoid metabolites and genes in the rice *rel1*-D mutant

**DOI:** 10.1186/s12864-025-11977-0

**Published:** 2025-09-01

**Authors:** Xiaojie Wu, Lingfang Yang, Jinbo Han, Hanqing Liu, Gaokun Chen, Haoyuan Wang, Xingru Feng, Wan Zhang, Kangping Liu, Zemin Zhang

**Affiliations:** 1 State Key Laboratory for Conservation and Utilization of Subtropical Agro‑Bioresources, Guangdong Provincial Key Laboratory of Plant Molecular Breeding, College of Agriculture, South China Agricultural University, Guangzhou, 510642 China; 2 Guangdong Yueliang Seed Industry Co., Ltd, Zhanjiang, China

**Keywords:** Transcriptome, Metabolome, *REL1*, Flavonoids, Heat-tolerence

## Abstract

**Background:**

Plants have evolved the ability to produce specialized metabolites as a defense mechanism against biotic and abiotic stressors, with flavonoid-mediated defense responses playing a crucial role in this process. Diverse flavonoids are present in various rice-grown resources, and they confer tolerance to different environmental conditions, including high temperature stress. Elucidating the differences in these flavonoids is essential for breeding improved rice varieties with enhanced tolerance to adverse environments. In a previous study, we isolated a dominant rice mutant generated by T-DNA insertion and christened it rolled and erect leaf 1 (hereafter *rel1*-D), initially identified for its enhanced tolerance to drought stress and its involvement in the regulation of leaf rolling and erectness. In this study, we utilized ZH11 and the *rel1*-D mutant as experimental materials to compare the expression profiles of genes and metabolites involved in the flavonoid pathway and high-temperature tolerance.

**Result:**

In our previous study, we generated a dominant mutant *rel1*-D in the ZH11 rice background via T-DNA insertion. Upon exposure to high-temperature stress followed by a recovery period, we observed that all ZH11 plants succumbed to the stress, whereas nearly 50% of the *rel1*-D mutants survived. Comprehensive transcriptomic and metabolomic analyses revealed 1,184 differentially expressed genes (DEGs) and 126 differentially abundant metabolites (DAMs) between the two genotypes. Notably, the majority of these differentially expressed genes and metabolites were enriched in the phenylalanine and flavonoid biosynthetic pathways in the *rel1*-D mutant. Specifically, the expression levels of key genes involved in flavonoid biosynthesis, including *OsCHI*,* OsF3H*,* OsFLS*,* OsCHS*,* OsPAL*, and *Os4CL*, were significantly upregulated in *rel1*-D, resulting in elevated levels of flavonoid compounds. Furthermore, we constructed a correlation network integrating phenotypic traits with the identified genes and metabolites. Our analysis indicated that the metabolism of flavonoids and phenolic compounds in leaves was positively correlated, whereas both were negatively correlated with yield-related traits.

**Conclusion:**

Potential genes regulated by ROLLED AND ERECT LEAF1 (*REL1)* and flavonoid metabolites were identified. *REL1* may affect the accumulation of flavonoid metabolites by regulating the expression of key genes in the flavonoid biosynthesis pathway to influence the heat tolerance of rice.

**Supplementary Information:**

The online version contains supplementary material available at 10.1186/s12864-025-11977-0.

## Background

Rice (Oryza sativa) is one of the world’s most important cereal crops, serving as a staple food for over half of the global population [1, 2]. Its growth, productivity, and quality are significantly influenced by environmental factors, with temperature being a critical determinant. Temperature fluctuations, especially high-temperature events, can have detrimental effects on rice development. Statistical data indicates that when the maximum daily temperature exceeds 38℃, it is classified as high temperature, which poses a significant threat to rice growth and development [1, 2]. High temperatures can lead to reduced yield, poor grain quality, and increased susceptibility to diseases and pests.

To mitigate the adverse effects of high temperatures and other environmental stresses, plants have evolved various physiological and biochemical mechanisms. One such mechanism involves the production of secondary metabolites, such as flavonoids. Flavonoids are a diverse group of phenolic compounds that play crucial roles in plant development, stress resistance, and overall plant health [3–5]. These compounds are synthesized through the phenylpropanoid pathway, one of the largest families of polyphenolic secondary metabolites found in plants [6]. The six primary classes of flavonoids include isoflavones, flavonoids, flavanones, flavonols, and anthocyanins [7, 8]. Flavonoids have been extensively studied for their biological activities and functions. They are known to act as antioxidants, scavenging reactive oxygen species (ROS) and protecting plants from oxidative damage [3, 9–13]. Additionally, flavonoids contribute to plant defense against biotic and abiotic stresses, such as drought, thermal stress, and pathogen infection [14]. Their presence can also deter herbivory by animals, thereby enhancing plant survival and reproductive success.

Beyond their roles in plant biology, flavonoids offer substantial nutritional and physiological benefits to humans. They possess antiviral, anticancer, anti-allergic, and anti-inflammatory properties and contribute to the prevention of coronary heart disease [15, 16]. Given these multifaceted benefits, enhancing the levels of flavonoids in rice could not only improve crop resilience but also enhance its nutritional value for human consumption.

In recent years, research has focused on understanding the stress resistance mechanisms of flavonoids, particularly their ability to withstand high temperatures, drought, and other environmental challenges [4, 5]. This knowledge is crucial for optimizing rice yield and quality under changing climatic conditions and for developing rice varieties with enhanced stress tolerance and nutritional profiles.

As of now, more than 9,000 unique flavonoid compounds have been recognized in a range of plant species [17]. Rice serves as a vital staple food, playing a crucial role in human nutrition. Additionally, it is often utilized as a model organism for studying monocotyledonous plants [18]. In contrast to dicotyledones such as Arabidopsis and Tomato [19], the biosynthetic pathways of flavonoids in rice are relatively unknown. Flavonoids are synthesized from L-Phenylalanine through the phenylpropanoid pathway, while L-Phenylalanine is produced via the shikimic acid pathway, as highlighted by several foundational studies [11, 20–22]. The initial three stages of the phenylpropanoid pathway are commonly known as the general phenylpropanoid pathway [23]. The role of *PAL* is essential in enabling the movement of carbon from primary to secondary metabolism in plants [24]. The initial two stages of the flavonoid biosynthetic pathway are characterized by the activities of chalcone synthase (*CHS*) and chalcone isomerase (*CHI*) [25]. These enzymes catalyze the production of chalcone, which subsequently results in the creation of flavanones, including naringin [26]. In rice, flavanones undergo conversion to their corresponding flavones through the action of either *OsFNS I-1* or *OsFNS II*, occurring in both laboratory settings and within the organism itself [25, 27, 28]. Dihydrokaempferol is produced via the enzymatic activity of flavanone 3-hydroxylase (*F3H*) [29]. The Flavonol synthase (*FLS*) facilitates the transformation of dihydroflavonols into flavonols via a desaturation process [30]. The *OsFLS* gene in rice encodes a dioxygenase that serves dual functions, demonstrating both flavonol synthase (*FLS*) and flavanone 3-hydroxylase (*F3H*) activities. It is essential in the production of flavonols [31]. In standard plant cells, approximately 20% of the total carbon flow is attributed to the flavonoid biosynthetic pathway [19]. The metabolic diversity of flavonoids in plants is crucial for their adaptation to a variety of environmental conditions.

The structural diversity of flavonoids significantly influences their biological activities. The capacity to gather stocks of metabolites unique to a species has been rapidly enhanced with the advent of metabolomics [32]. After the successful sequencing of the rice genome in 2005, rice has become a preferred model organism in the field of functional genomics, primarily because of its comparatively small genome size of around 389 Mb [33]. A thorough and systematic identification and analysis of the essential structural genes involved in the biosynthesis of flavonoid scaffolds remains to be comprehensively undertaken, apart from those associated with the CHS gene families [34, 35]. The rolled and erect leaf1 (*rel1*-D) mutant is a dominant rice mutant initially identified for its role in regulating leaf rolling and erectness. This mutant was generated through T-DNA insertion and exhibits phenotypes characterized by leaf rolling and erect growth. Research has demonstrated that *rel1*-D displays enhanced tolerance under drought stress conditions, with significantly upregulated superoxide dismutase (SOD) activity and expression of drought-responsive genes. Notably, rel1-D also exhibits hypersensitivity to abscisic acid (ABA), with a marked increase in the expression of ABA-related genes. These findings suggest that *REL1* may modulate drought responses by coordinating ABA signaling.Further transcriptomic analyses have revealed a substantial number of differentially expressed genes (DEGs) in *rel1*-D, primarily associated with metabolic changes and stress responses. These DEGs show significant enrichment in cellular components, molecular functions, and biological processes, particularly in cell wall, external encapsulating structures, cell periphery, hydrolase activity, transcription factor activity, and catalytic activity. Additionally, the ABA signaling pathway is significantly altered in *rel1*-D, further supporting the role of ABA in *REL1*-mediated leaf morphology and stress responses.The study of *rel1*-D not only enhances our understanding of the physiological mechanisms underlying rice responses to drought stress but also provides a crucial theoretical foundation for improving crop stress resistance through genetic regulation.This study aims to fill the current knowledge gap regarding the biosynthetic pathways of flavonoids in rice. Although previous research has explored the role of flavonoids in plant stress resistance, systematic studies specifically targeting rice are still limited. In particular, the key genes and regulatory mechanisms involved in the biosynthetic pathways of flavonoids in rice remain largely unexplored. Additionally, this study will integrate transcriptomics and metabolomics data to elucidate the specific mechanisms by which flavonoids contribute to rice growth and stress responses, an area that has not been fully investigated in previous studies. Therefore, this study not only enhances our understanding of the biosynthetic pathways of flavonoids in rice but also provides a theoretical basis for breeding rice varieties with stronger stress resistance.

## Results

Striking morphological disparities were evident between the two varieties examined. Relative to the wild-type ZH11 (WT), the *rel1*-D mutant displayed a suite of distinctive traits, including leaf rolling, reduced plant stature, shorter panicle length, decreased tiller number, and narrower leaf width. Notably, both the leaf roll index and leaf bending were markedly elevated in *rel1*-D, with statistical significance (*p* < 0.05). In contrast, parameters such as maximal leaf width, 10-grain width, 10-grain length, and 100-grain weight remained largely unchanged [36].

### Phenotype analysis of heat stress in rice seedlings

**Fig. 1:**
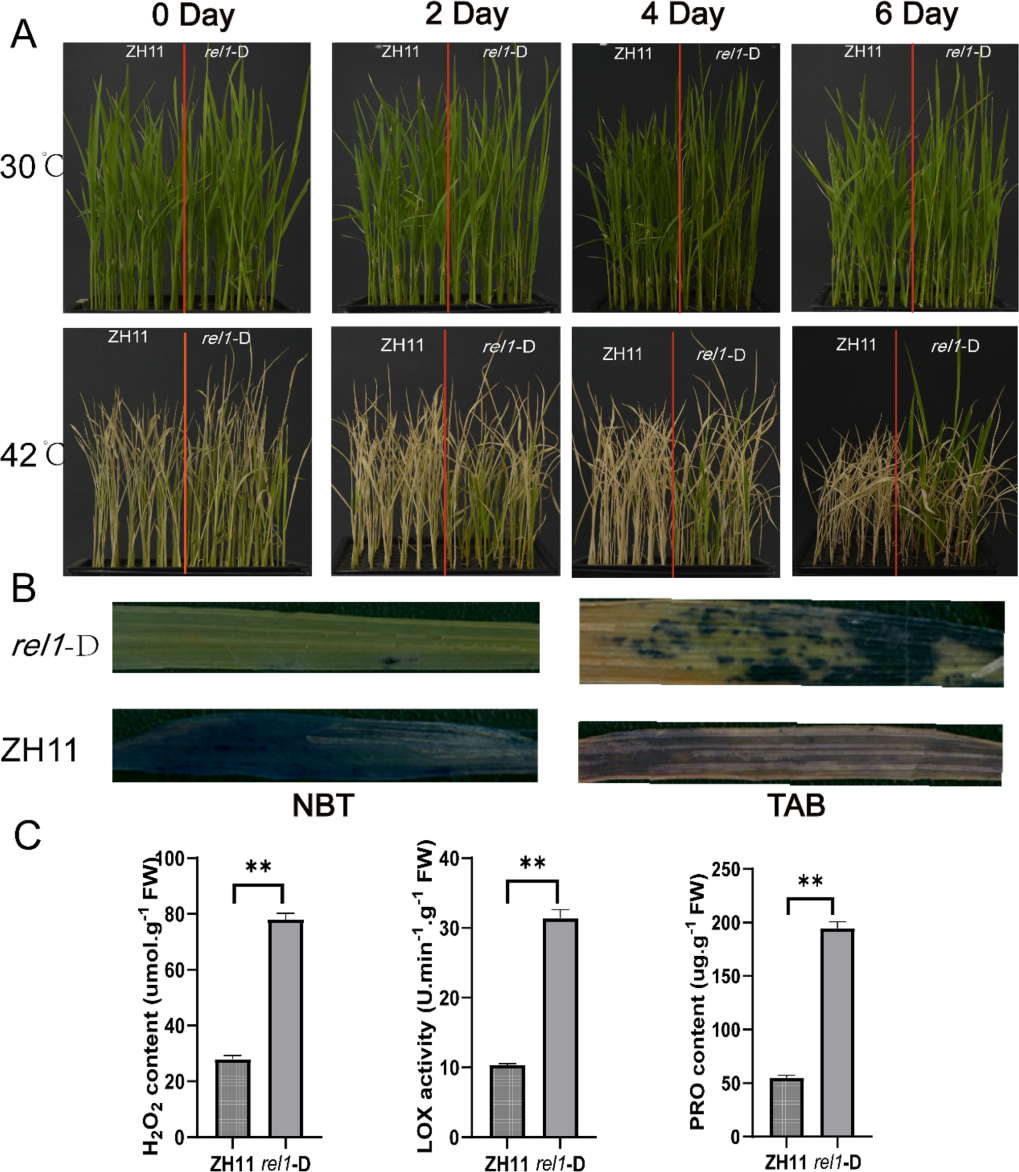
Phenotypes of ZH11 and *rel1*-D mutants under high temperature treatment. (A), 30℃ is thenormal growth temperature, 42℃ represents high temperature treatment, and 0,2,4,6 days represent the time of returning to normal temperature after heat stress. (B), NBT and TAB staining were performed on the materials recovered on the 0 day. (C), The levels of intracellular hydrogen peroxide, as well as lipoxygenase and proline content, in ZH11 and *rel1*-D at day 0 of recovery (*p* < 0.05)

The regreening phenotype of ZH11 and *rel1*-D rice seedlings under high temperature (42 ℃) was shown in Fig. 1A. After a 48 h high-temperature stress, no notable differences in appearance were seen between ZH11 and *rel1*-D. The survival of the *rel1*-D mutant leaf was observed to increase with the extention of recovery time, while ZH11 was dead on the 6th day (Fig. S1), which indicated that *rel1*-D could tolerate high temperature and recover from heat damage.

In this study, NBT staining revealed that superoxide anion was released in both ZH11 and *rel1*-D cells immediately following exposure to high-temperature stress (Fig. 1B). This finding indicates that both cell types were under oxidative stress, suggesting that high-temperature stress had already triggered oxidative reactions within the cells. Conversely, TAB staining showed that all ZH11 cells died after 0 days of high-temperature stress, whereas cell death was significantly reduced in *rel1*-D (Fig. 1B).

In addition, compared with ZH11, the levels of hydrogen peroxide (H_2_O_2_), proline (PRO), and lipoxygenase (LOX) were significantly higher in the *rel1*-D mutant (Fig. 1C). Hydrogen peroxide (H_2_O_2_) is a reactive oxygen species (ROS) that acts both as a product of oxidative stress and as an important signaling molecule in plant cells. The elevated levels of H_2_O_2_ in the *rel1*-D mutant may reflect its enhanced antioxidant capacity. This is because high levels of H_2_O_2_ can act as a signaling molecule to induce the expression of antioxidant enzymes, such as catalase and superoxide dismutase, thereby enhancing the cell’s tolerance to oxidative stress. Proline is an important osmoprotectant that helps maintain cellular osmotic pressure and protect cellular structure and function under stress conditions. Additionally, proline can act as an antioxidant by scavenging free radicals to mitigate oxidative damage. The significant increase in proline content in the *rel1*-D mutant helps maintain cellular osmotic balance and antioxidant capacity under high-temperature stress.Lipoxygenase (LOX) is involved in the protective mechanisms of plant cell membranes. It catalyzes the oxidation of fatty acids to produce protective metabolites. Moreover, increased LOX activity can enhance membrane fluidity and stability. The significant increase in LOX activity in the *rel1*-D mutant helps maintain membrane integrity, thereby reducing membrane damage under high-temperature stress.

Collectively, the results from NBT staining, TAB staining, and enzyme activity assays demonstrate that *rel1*-D possesses a stronger antioxidant capacity than ZH11. These findings reveal that the *rel1*-D mutant significantly enhances its heat tolerance under high-temperature stress by upregulating antioxidant defense mechanisms, including the increased expression and activity of H_2_O_2_, PRO, and LOX.

### Metabolome analysis

**Fig. 2:**
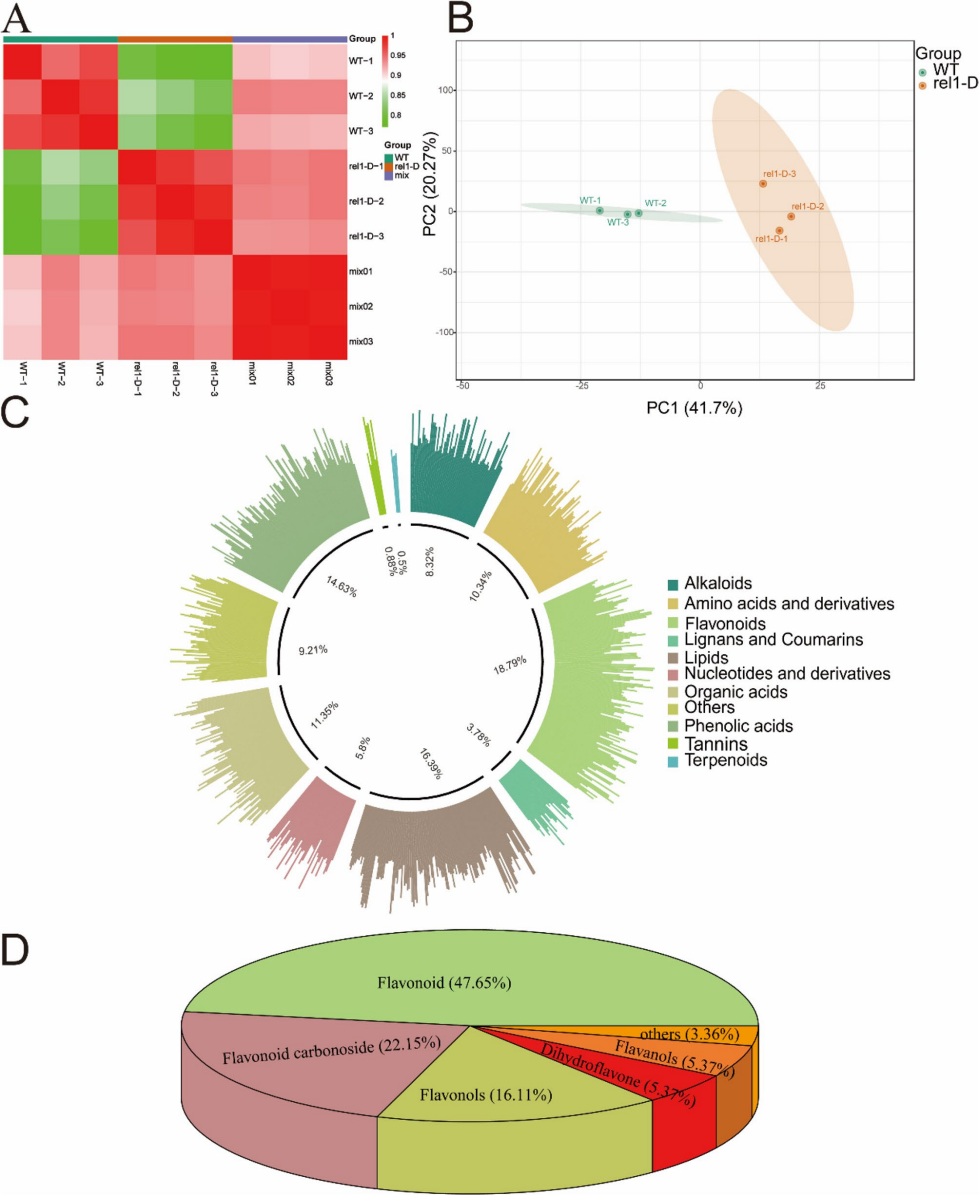
Metabolite Profiles in Wild-Type and Mutant Rice. **A** Pearson correlation analysis between wild-type (WT) and rel1-D mutant replicates. The x-axis and y-axis represent the sample names, with each axis corresponding to the same set of samples. The color gradient reflects the magnitude of the correlation coefficients, ranging from low (cool colors) to high (warm colors). Sample grouping is indicated by the “Group” label. **B** Principal Component Analysis (PCA) of wild-type (WT) and rel1-D mutant samples. PC1 represents the first principal component, and PC2 represents the second principal component. The percentages indicate the proportion of variance explained by each principal component within the dataset. Each point on the plot represents a sample, and samples from the same group are indicated by the same color. The grouping of samples is labeled as “Group.” **C** Comparative abundance of different metabolites. The percentages indicate the proportion of each metabolite within the respective superclasses. **D** Proportion of each subclass of flavonoid metabolites. The percentages indicate the proportion of each flavonoid metabolite relative to the total metabolite pool, with each color representing a distinct flavonoid metabolite.

Using a targeted metabolomics approach, we identified 793 metabolites across two groups. The excellent reproducibility of the analysis was demonstrated by the analysis of three biological replicates per sample (Fig. 2A). Principal Component Analysis (PCA), a widely used dimensionality reduction technique, was employed in this study. Here, 528 hybrid combinations of metabolites were classified into PCA1 (41.7%) and PCA2 (20.27%), which together account for 61.97% of the total variance (Fig. 2B). This indicates that PCA1 and PCA2 are the most significant dimensions in the data.

The distribution of metabolites along PCA1 and PCA2 revealed distinct clustering patterns. These patterns suggest that different metabolites have varying contributions to the two principal components, thereby forming distinct groups. Overall, through PCA and clustering analysis, the complex metabolite data can be simplified into more interpretable patterns, providing crucial insights into the biological functions and metabolic regulation mechanisms of the identified metabolites. Among them, 82 of them, 116, 46, 149, 30, 7, 66, and 73 (Fig. 2C, Table S1). It was found that flavonoids, lipids and phenols were the most abundant. The flavonoid category includes chalcones, dihydroflavone, dihydroflavonol, flavanols, flavonoids, flavonoid carbonoside, and flavonols, with flavonoids making up 47.65% of the total (Fig. 2D).

**Fig. 3:**
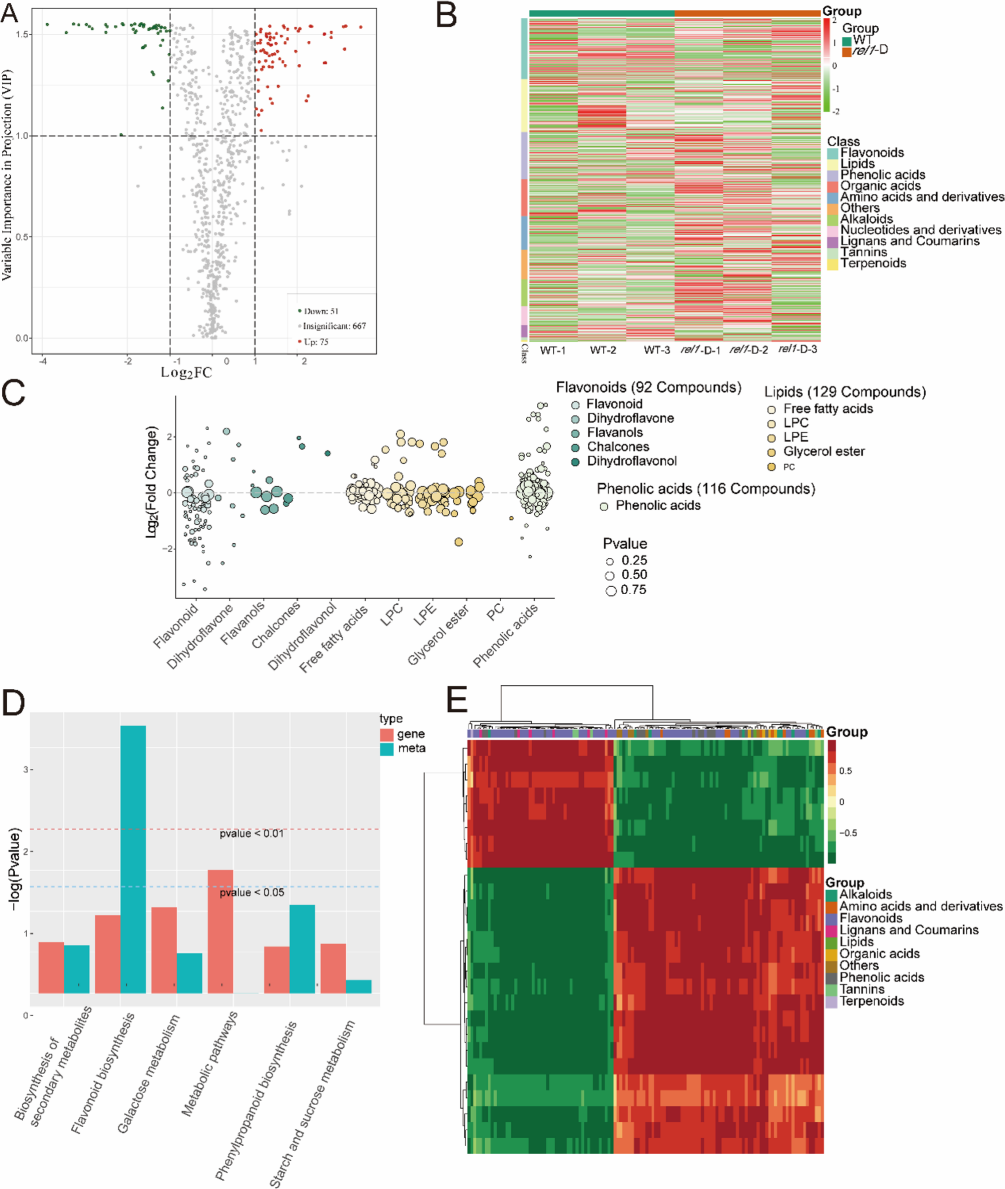
Integration Analysis of Differential Metabolites and Differential Genes. **A** Volcano plot of DAMs. Each point in the plot represents an individual metabolite. The x-axis displays the logarithm of the fold change in metabolite abundance between the two samples, while the y-axis shows the VIP (Variable Importance in Projection) value. Metabolites are color-coded according to their expression patterns: green points indicate downregulated metabolites, red points indicate upregulated metabolites, and gray points represent metabolites that were detected but did not exhibit significant differences. **B** Proportional representation of DAMs. The x-axis lists the sample names, while the y-axis provides detailed metabolite information. Samples are grouped and labeled accordingly as “Group,” and metabolites are classified and denoted as “Class.” The heatmap uses a color gradient to represent normalized relative abundance values, with red indicating high abundance and green indicating low abundance. **C** Classification plot of differential metabolites. The x-axis lists the differentially expressed metabolites, while the y-axis indicates their corresponding log2 fold changes (log2FC). **D** Pathway enrichment analysis of DAMs and differentially expressed genes (DEGs). The x-axis represents the pathway maps. On the y-axis, red bars indicate the enrichment p-values for DEGs, while green bars represent the enrichment p-values for DAMs. These values are presented as –log(P-value), with higher values on the y-axis corresponding to stronger enrichment significance. **E** Correlation analysis between DAMs and DEGs

The metabolic changes of mutant *rel1*-D and ZH11(WT) were studied by metabolomics. In this paper, the differential accumulation of DAMs (DAMs) was investigated in the tillering period (Fig. 3A). A total of 126 metabolites were identified as being differentially expressed, with 75 displaying increased levels and 51 demonstrating decreased levels (refer to Fig. 3B and Table S2). Co-association KEGG enrichment analysis revealed that *rel1*-D co-maps with ZH11(WT) in six pathways (Table S3), and interestingly, among these synthetic pathways, the flavonoid pathway and phenypropanoid biosynthesis were highly enriched (Fig. 3C, D). The findings indicated a positive relationship between these genes and the regulation of metabolites, implying that these genes could have a beneficial effect on gene regulation (Fig. 3E). The highly correlated DAMs and DEGs were shown in the heat map. 

### Variations in gene expression between two materials

Transcriptome sequencing was performed on six samples, resulting in a total of 39.04 GB of clean data, with each sample providing more than 6.14 GB. The Q30 rate exceeded 94.49% (Table S4). The processed reads from each sample were mapped to the specified reference genome, yielding alignment rates between 87.72% and 96.27% (see Table S5).

**Fig. 4:**
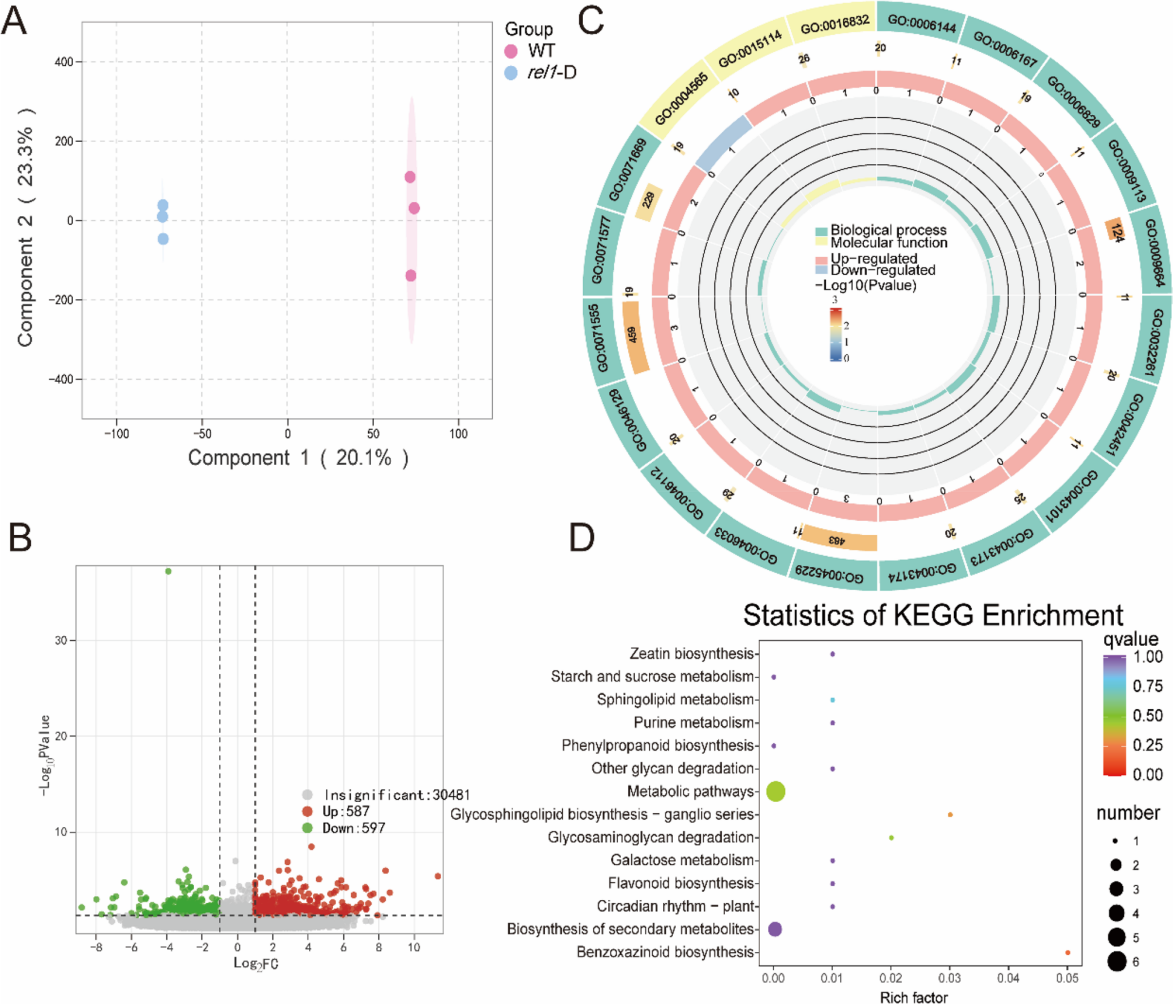
Transcriptome Profiles of Wild-Type and Mutant Species. **A** Principal Component Analysis (PCA) of the transcriptome. PC1 represents the first principal component, and PC2 represents the second principal component. The percentages indicate the proportion of variance explained by each principal component within the dataset. Each point on the plot represents a sample, and samples from the same group are indicated by the same color. The grouping of samples is labeled as “Group.” **B** Volcano plot of the transcriptome. The x-axis represents the fold change in gene expression, while the y-axis indicates the significance level of differentially expressed genes. Red points denote upregulated differentially expressed genes, green points represent downregulated differentially expressed genes, and gray points indicate genes that are not differentially expressed. **C** Gene Ontology (GO) enrichment analysis. **D** Kyoto Encyclopedia of Genes and Genomes (KEGG) enrichment analysis. Pathways are displayed along the vertical axis, while the Rich factor is displayed along the horizontal axis. A higher Rich factor indicates a greater level of enrichment, suggesting a larger number of associated genes, more significant differences, and a higher concentration of gene functions within the pathway

The Cluster Analysis Heatmap showed that there was a strong bioreproducibility in all three varieties. This study identified 1,184 differentially expressed genes (DEGs), consisting of 337 with elevated expression levels and 280 with reduced expression levels (Fig. 4A, B and Table S6). The GO annotation analysis revealed that 16 DEGs were categorized into various biological processes and molecular functions (Fig. 4C), with a significant proportion associated with molecular and metabolic processes. Among the molecular processes, hydrolases were the most prevalent. KEGG enrichment analysis demonstrated a significant association between differentially expressed genes (DEGs) and both the flavonoid biosynthesis pathway and the biosynthesis of secondary metabolites (Fig. 4D and Table S7). The pronounced enrichment of the flavonoid biosynthesis pathway in our KEGG analysis highlights that genes within this pathway exhibited substantial expression changes under the experimental conditions. Similarly, the significant correlation between DEGs and the biosynthesis of secondary metabolites pathway indicates that genes within this pathway also underwent marked expression alterations. Collectively, these results suggest that the *rel1*-D mutant exerts a pronounced influence on the flavonoid biosynthesis pathway, likely through the modulation of relevant gene expression, thereby affecting the synthesis and accumulation of flavonoids.

### qRT-PCR was employed to confirm the transcriptome data

**Fig. 5:**
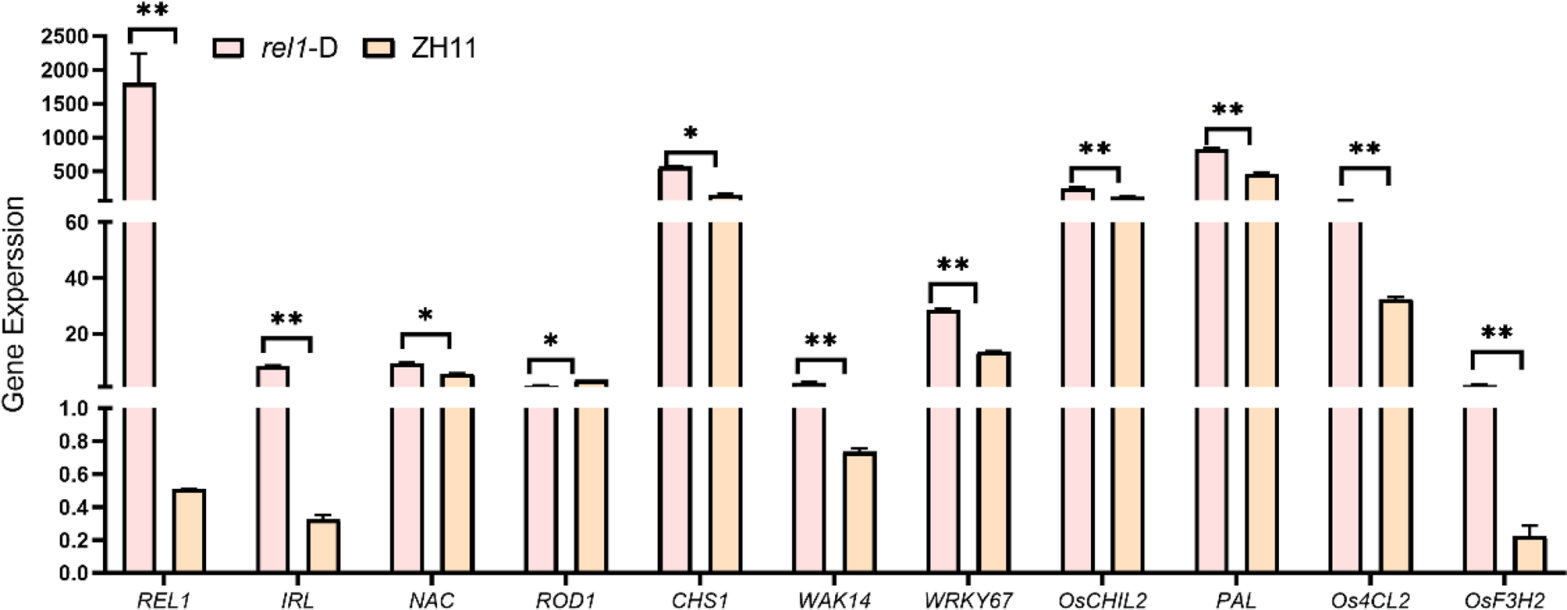
qRT-PCR of several genes

To verify the outcomes of the RNA-Seq analysis, qRT-PCR was conducted to corroborate the transcriptomic results. A selection of random genes, including *OsREL1*, *OsIRL*, *OsNAC4*,* OsROD1*,* OsCHS1*,* OsWAK14*,* OsWRKY67*,* OsPAL*,* OsCHIL2*,* Os4CL2*, and *OsF3H2*, were analyzed in the tillering leaves of ZH11 and *rel1*-D (Fig. 5). The findings demonstrated that there was no notable difference between the results obtained from RNA-Seq and qRT-PCR, suggesting a uniform pattern across both techniques. The RNA-Seq data utilized in this study was confirmed for its reliability.

### Coexpression networks demonstrate varying regulation of important genes, metabolites, and physiological characteristics

A co-expression network was established to explore the possible interactions among genes associated with flavonoid production. This network comprised 581 genes along with 126 differentially expressed transcription factors, as measured by Pearson correlation coefficients (PCCs). The selection of the β value is essential to achieve a scale-free network topology, where the degree distribution follows a power-law distribution. This means that a few nodes (hubs) have many connections, while most nodes have relatively few connections. In this study, we analyzed the scale-free topology fit index (R²) and mean connectivity for various β values. We found that when β = 5, the network’s scale-free topology fit index (R²) reached 0.85, and the mean connectivity stabilized. This indicates that the network exhibits good scale-free properties at this β value, making it an optimal choice for constructing a biologically meaningful gene co-expression network [37]. After the optimum parameter (β = 5) was determined, the WGCNA algorithm (Fig. S2 A, B) was used to transform the correlation coefficients of each gene pair into neighboring coefficients. In the next phase, we performed WGCNA to further clarify the connection between gene expression patterns and the biosynthesis and accumulation of flavonoids. The genes were categorized into 11 separate modules, and the Pearson correlation coefficient was employed to assess the relationships among genes across various modules. The intensity of the color reflects the strength of the module’s correlation (see Figures S2 C and S3). Therefore, the dark module could be used to predict the coexpression network of flavonoids in rice.

**Fig. 6:**
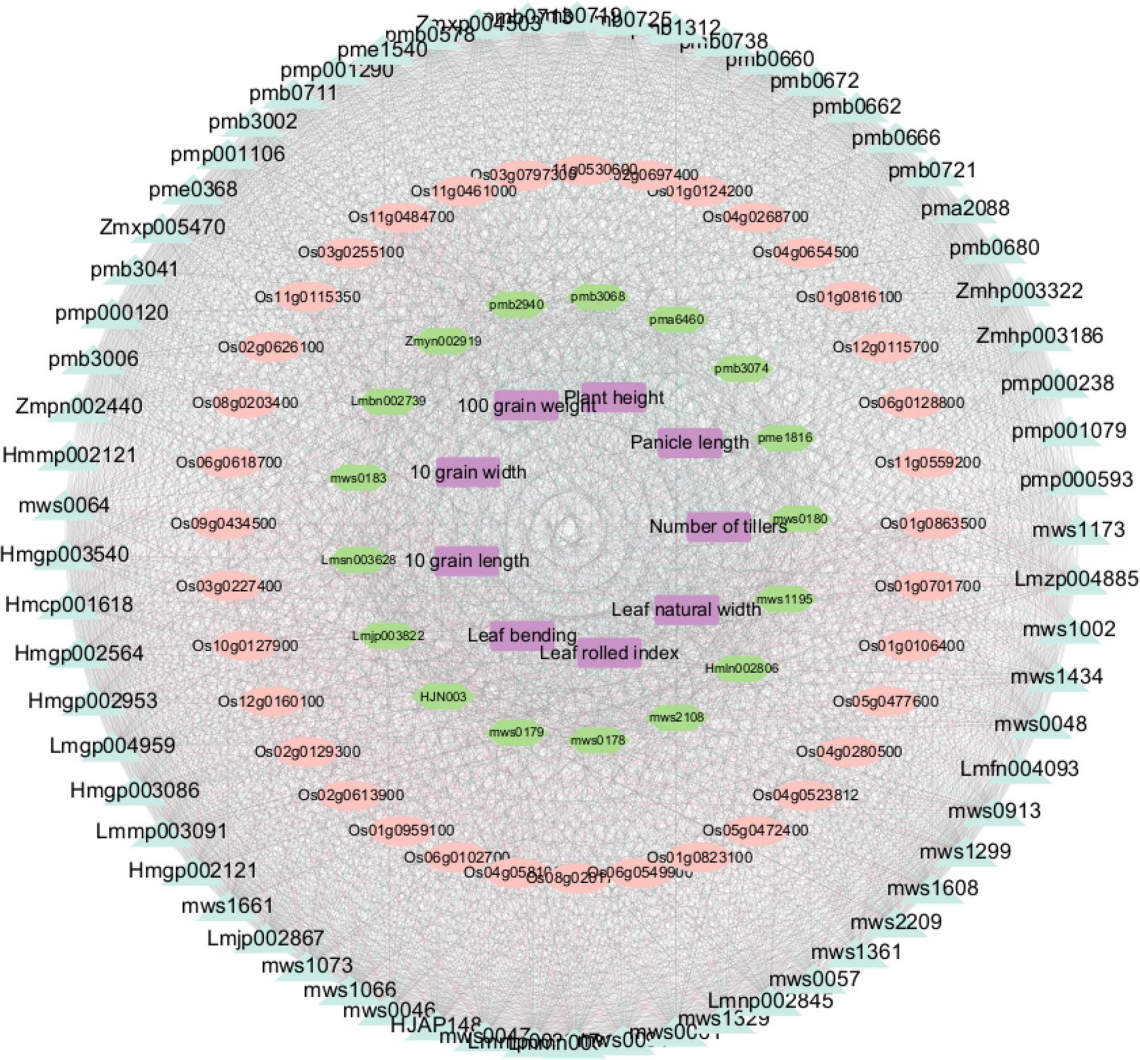
The potential regulatory networks of genes and metabolites involved in flavonoid biosynthesis pathway, as well as various physiological characteristics. The coloured nodes represent the biosynthesis of flavonoids (blue), the metabolites of phenol (green) and the physiological properties (violet)

Based on Pearson correlation analysis, we constructed a complex regulatory network encompassing key genes, metabolites, and physiological traits of the mutants. This analysis focused on 36 genes, 82 metabolites, and 10 physiological traits (for details, see Table S8). Utilizing Cytoscape software, we visualized the regulatory network pairs with a Pearson correlation threshold greater than 0.8 (see Fig. 6). The analysis revealed a network comprising 128 nodes connected by 3,884 edges, elucidating the intricate associations between genes and metabolites. Specifically, 1,840 genes exhibited negative correlations, while 244 genes showed positive correlations. Overall, a significant positive relationship was observed between the metabolism of flavonoids and phenols and the gene expression in leaves. Flavonoids and phenols play indispensable roles in plant growth, development, and stress responses. They are not only efficient antioxidants but also participate in defense against pests and diseases and adaptation to various environmental stresses. Our in-depth analysis of the regulatory network revealed that these metabolic pathways are tightly linked to gene expression in leaves. This finding suggests that the identified modules may play a crucial role in regulating the synthesis and accumulation of flavonoids and phenols.

The complex connections of 128 nodes and 3,884 edges in the network reveal a high degree of co-regulation between gene expression and metabolite accumulation. Leaves, as the primary organs for photosynthesis and metabolic activities in plants, show a positive correlation between gene expression and flavonoid and phenol metabolism, which is closely related to leaf physiological functions. For example, leaf morphological traits (such as leaf rolling) may interact with metabolite accumulation, thereby affecting the overall physiological state of the plant. This co-regulation mechanism provides an important physiological basis for plant survival and adaptation under stress conditions. Flavonoids and phenols are crucial for plant stress responses. By regulating these metabolic pathways, plants can significantly enhance their antioxidant capacity and stress resistance. The identified modules may play a key role in helping plants cope with high temperatures, drought, and other stress conditions by regulating gene expression and metabolite accumulation to enhance adaptability. This discovery not only provides a new perspective for understanding plant stress response mechanisms but also offers potential targets for breeding crop varieties with stronger stress resistance. Key genes in the network may encode transcription factors or signaling proteins that play a central role in regulating metabolic pathways. Further investigation of these genes’ functions can reveal their regulatory mechanisms in plant growth and stress responses. These studies not only deepen our understanding of plant biological processes but also provide important theoretical support for genetic improvement. Through in-depth discussion and analysis of the identified modules, we have revealed the regulatory relationships between flavonoid and phenol metabolism and gene expression and explored the potential roles of these modules in plant physiological functions and stress responses. These findings provide an important theoretical foundation for future research and point the way for genetic improvement in rice. We look forward to further studies that will uncover the functions of these modules and provide strong support for breeding rice varieties with greater stress resistance and higher yields.

### DEGs and DAMs in the biosynthesis of flavonoids

**Fig. 7:**
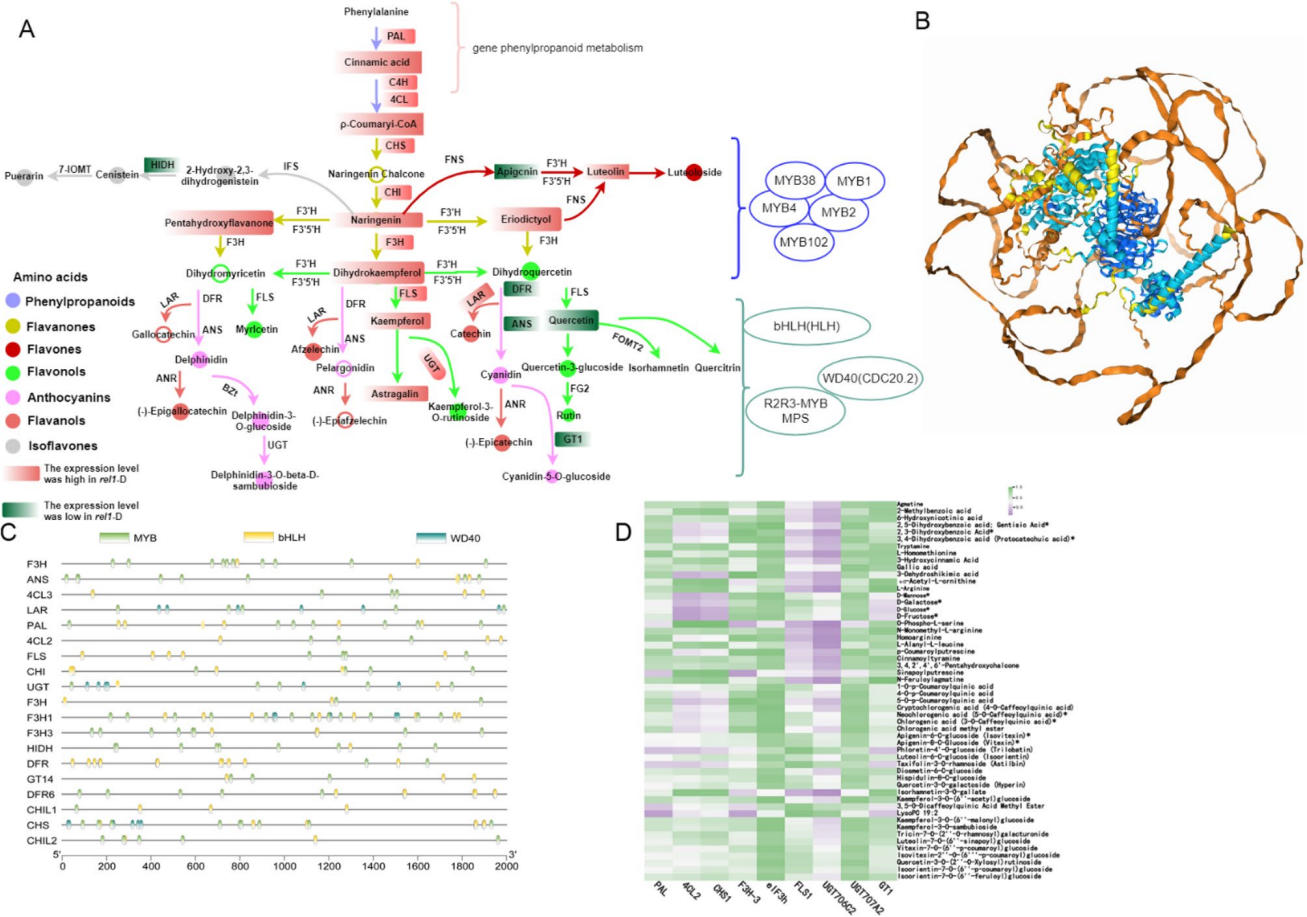
Gene, Metabolite, and Transcription Factor Co-regulation Analysis. **A** Analysis of genes, metabolites, and transcription factors in the flavonoid pathway. Lines represent enzymatic chemical reactions, while circle nodes represent metabolites. The color of a solid circle (indicating detection) or a hollow circle (indicating non-detection) signifies the type of metabolite. The enzymes discussed in this paper include Phenylalanine ammonia-lyase (*PAL*), 4-coumarate CoA ligase (*4CL*), chalcone synthase (*CHS*), chalcone isomerase (*CHI*), flavanone 3-hydroxylase (*F3H*), flavonoid 3′-hydroxylase (*F3′H*), flavonol synthase (*FLS*), dihydroflavonol reductase (*DFR*), anthocyanidin reductase (*ANR*), and leucoanthocyanidin reductase (*LAR*). Red squares represent increased expression levels in *rel1*-D, while green squares indicate decreased expression levels. Different colored circles represent different classes of metabolites: for example, purple indicates phenylpropanoids, yellow indicates flavanones, red indicates flavones, green indicates flavonols, pink indicates anthocyanins, light red indicates flavanols, and gray indicates isoflavones. **B** Protein interaction model of *OsHLH*,* OsMPS*, and *OsCDC 20.2* predicted by AlphaFold3. **C** Cis-acting elements located in the promoters of DEGs involved in flavonoid biosynthesis. **D** Correlation analysis among key genes (*PAL*,* C4H*,* 4CL*,* CHS*,* CHI*,* F3H*,* FLS*) and metabolites (flavanone, flavanone, flavonol) in the flavonoid pathway, with a Pearson correlation coefficient of ≥ 0.9 and a p-value of ≤ 0.05. The x-axis represents genes, while the y-axis represents metabolites

Through the implementation of KEGG pathway enrichment and gene ontology functional analysis, we identified 21 DEGs that code for enzymes participating in the biosynthesis of flavonoids and phenolic acids. This included 1 *OsPAL* DEG, 1 *OsC4H* DEG, 2 *Os4CL* DEGs, 2 *OsCHS* DEGs, 4 *OsCHI* DEGs, 3 *OsF3H* DEGs, 1 *OsFLS* DEG, 2 *OSUGT* DEGs, 1 *OsDFR* DEG, 1 *OsANS* DEG, 1 *OsLAR* DEG, 2 *OSGT1* DEGs, and 1 *OsHIDH* DEG. To explore the mechanisms that contribute to flavonoid accumulation in rice, we analyzed the expression patterns of genes linked to the phenylpropanoid and flavonoid biosynthetic pathways. The phenylpropanoid pathway generates crucial precursors necessary for the synthesis of flavonoids, such as phenylalanine. The initial steps in the flavonoid pathway are facilitated by the enzymes *PAL* and *CHS*, which are succeeded by a series of downstream reactions catalyzed by different enzymes, ultimately leading to the production of aglycones (see Fig. 7A). The increased expression of *OsPAL*,* OsC4H* and *Os4CL* guarantees a sufficient availability of precursors necessary for flavonoid biosynthesis, especially considering the vital role of *Os4CL* in the synthesis of p-coumaroyl-CoA. This observation could elucidate the increased concentrations of flavanones, flavones, flavonols, isoflavones, and flavanols identified in *rel1*-D. Flavonoids were produced from naringenin, which was formed via the enzymatic activity of *CHI.* In this research, we noted that the increased expression of *OsCHI* in the *rel1*-D variant resulted in the greatest accumulation of naringenin. Furthermore, an increased presence of downstream compounds such as flavones, flavonols, isoflavones, and flavanols was observed in *rel1*-D, which is likely associated with the upregulation of *OsF3H* genes in this particular variant.

The transcription factors OsMYB1, OsMYB2, OsMYB4, OsMYB38, OsMYB102, OsHLH, OsMPS, OsDCD 20.2 were highly expressed in *rel1*-D. Predictions from the AlphaFold model (https://golgi.sandbox.google.com/) showed that the protein structures of transcription factors OsHLH, OsMPS and OsCDC 20.2 interacted (Fig. 7B). OsHLH (OsLHW), a bHLH-class transcription factor, is implicated in rice root development and drought stress response. While the primary function of OsHLH is associated with drought stress, the bHLH transcription factor family is known to play a significant role in plant responses to various abiotic stresses. Given this, OsHLH may similarly regulate the expression of related genes under high-temperature stress, thereby enhancing plant thermotolerance. OsMPS, an R2R3-type MYB transcription factor, integrates multiple hormone pathways to regulate plant adaptive growth. MYB transcription factors are crucial for plant thermotolerance. OsMPS may enhance plant thermotolerance under high-temperature stress by regulating the expression of hormone signaling pathway genes and cell wall synthesis-related genes. OsCDC20.2 is a cell cycle-related gene that regulates the mitotic process of plant cells. OsCDC20.2 may help plants maintain normal cell division and growth under high-temperature stress by regulating the expression of cell cycle proteins, thereby enhancing plant thermotolerance. In summary, while OsHLH, OsMPS, and OsCDC20.2 play important roles in plant responses to drought, salt, and cold stresses, they may also enhance plant thermotolerance by regulating gene expression, hormone signaling pathways, and cell cycle processes. Among the promoters of *OsPAL*,* OsC4H*,* Os4CL*,* OsCHI*,* OsF3H*,* OsFLS*,* OsLAR*,* OsUGT*,* OsGT14*,* OsANS*,* OsDFR*, etc., several binding original promoters of MYB, bHLH, WD40 (Fig. 7C) were found, which indicated that these genes and transcription factors controlled the production of flavonoids in plants. The transcription levels of *OsFLS* were observed to be elevated in *rel1*-D, and the concentrations of myricetin, quercetin, kaempferol, astragalin, quercitrin, rutin, iso-hamnetin, quercetin 3-glucoside, and kaempferol 3-O-rutinoside in *rel1*-D were markedly higher in comparison to other stages. This study highlights the critical role of genes *OsPAL*,* OsC4H*,* Os4CL*, *OsCHS*,* OsCHI*,* OsF3H* and *OsFLS* in the biosynthesis of key flavonoid components, including flavanones, flavones, and flavonols, in the *rel1*-D variant. The high expression levels of these genes may help plants maintain normal growth and development under high-temperature stress, thereby enhancing their thermotolerance.

A Pearson correlation analysis was conducted to investigate the association between the expression levels of *OsPAL*,* OsC4H*,* Os4CL*,* OsCHS*,* OsCHI*,* OsF3H*, and *OsFLS*—essential enzymes involved in the flavonoid biosynthesis pathway—and the relative concentrations of 70 differentially expressed flavanones, flavones, and flavonols, as outlined in Table S8. A correlation coefficient of ≥ 0.8 and a significance level of *P* < 0.05 were utilized for this assessment [38]. The study identified a total of 12 DEGs related to various biosynthetic pathways. Specifically, four DEGs associated with *OsCHI* (*Os03g0132900*,* Os10g0416500*,* Os12g0115700*,* Os11g0116300*), three DEGs linked to *OsF3H* (*Os04g0376500*,* Os04g0667200*,* Os04g0662600*), one DEG for *OsFLS* (*Os02g0767300*), one DEG for *OsCHS* (*Os11g0530600*), one DEG for *OsPAL* (*Os02g0626100*), and two DEGs for *Os4CL* (*Os02g0697400*,* Os02g0177600*) were observed. The identified DEGs exhibited significant correlations with various compounds, such as dihydroquercetin, hyperoside, kaempferol, kaempferol-3-O-rutinoside, luteolin, luteoloside, myricetin, naringenin, nobiletin, quercetin, quercetin 3-glucoside, rutin, and tiliroside, as illustrated in Fig. 7D. The findings indicate that these nine DEGs are instrumental in the accumulation of beneficial components in the *rel1*-D plant variant.

## Discussion

The *rel1*-D mutant demonstrated a greater survival rate and a higher proportion of green phenotype compared to ZH11 (Fig. 1, Fig. S1). RNA-seq and metabolome analyses were employed to dissect the molecular mechanisms underlying the heat tolerance of two rice varieties at the tillering stage. These findings not only enhance our understanding of how rice regulates itself under abiotic stress but also lay a solid theoretical foundation for future breeding and selection of varieties capable of withstanding high temperatures.

### Flavonoids as pivotal players in stress tolerance

Flavonoids are essential secondary metabolites in plants, significantly contributing to various biological processes such as pathogen defense, growth regulation, and root development [37–42]. The *rel1*-D mutant exhibited notably elevated levels of several key flavonoids, including quercetin, kaempferol, and their derivatives, which are renowned for their antioxidant properties and ability to bolster plant resilience under stress condition-s [43, 44]. These flavonoids function not only as antioxidants, neutralizing reactive oxygen species (ROS) that accumulate under stress, but also as signaling molecules that activate downstream stress response pathways [45]. For instance, quercetin and kaempferol have been shown to enhance the expression of heat shock proteins (HSPs) and antioxidant enzymes, thereby fortifying the plant’s defense mechanisms against oxidative damage [46].

### Regulatory mechanisms of flavonoid biosynthesis

A multitude of enzymes involved in the biosynthesis of flavonoids have been identified, including *OsPAL*,* OsC4H*,* Os4CL*,* OsCHS*,* OsCHI*,* OsF3H*,* OsFLS*,* OsUGT*,* OsDFR*,* OsANS*,* OsLAR*,* OsGT1*, and *OsHIDH* [47–52]. In the *rel1*-D mutant, the expression levels of these genes were significantly upregulated, leading to a substantial increase in flavonoid production. Specifically, the elevated expression of *OsPAL*,* OsC4H*, and *Os4CL* ensures the availability of p-coumaryl-CoA, a crucial precursor for flavonoid synthesis. This, in turn, enhances the concentrations of downstream flavonoids such as flavonols, flavones, and dihydroflavonols.

The biosynthetic processes of dihydroflavonol reductase (DFR) and flavonol synthase (FLS) are particularly noteworthy, as they compete for the precursor dihydromyricetin (DHM) [53]. The increased transcription of the *OsFLS* gene in the *rel1*-D mutant suggests that the plant prioritizes the production of flavonols over anthocyanins, which may contribute to its enhanced stress tolerance [47–52]. This prioritization likely reflects an adaptive strategy where the plant allocates resources to produce metabolites with broader protective functions rather than those with more specialized roles [54].

### Gene expression patterns and tissue-specificity

The strong connection between flavonoids and plant adaptation to the environment was evident in the tissue-specific expression patterns of the genes examined in this study [55]. Certain genes, such as *OsANS*,* OsFLS1*, and *OsCHI*, exhibited markedly increased expression levels in the *rel1*-D mutant, suggesting that they play a significant role in the mutant’s enhanced stress tolerance. These findings align with previous research on other plant species [47–52] and highlight the importance of tissue-specific gene expression in stress response. For instance, the elevated expression of *OsCHI* in the leaves of *rel1*-D indicates that this gene may be specifically activated in response to heat stress, thereby enhancing the production of flavonoids in tissues most vulnerable to oxidative damage [56–61].

### Metabolite accumulation and physiological functions

A comprehensive metabolome analysis revealed a total of 793 metabolites, of which 83 were categorized as flavonoids. This group included 43 flavonoids, 15 flavonols, 2 chalcones, 4 dihydroflavones, and 1 dihydroflavonol. The levels of these flavonoids were significantly elevated in the *rel1*-D mutant, particularly chalcones, dihydroflavones, flavonoid carbonosides, flavonols, dihydroflavonols, and flavonoids. The elevated levels of specific flavonoids, such as quercetin-3-o-(2”-o-xylose), rutin, kaempferol-3-o-(6”-o-acetyl) glucoside, and kaempferol-3-o-(6”-malonyl) glucoside, suggest that they play a crucial role in the mutant’s resilience to both drought and heat stress [37, 44, 45].

These flavonoids likely function through multiple mechanisms. For example, quercetin and kaempferol can directly scavenge ROS, reducing oxidative damage to cellular components such as lipids, proteins, and nucleic acids [62]. Additionally, these compounds may modulate the activity of antioxidant enzymes, further enhancing the plant’s capacity to mitigate oxidative stress [63–65]. The elevated levels of these flavonoids in *rel1*-D suggest that they are integral to the mutant’s enhanced stress tolerance, providing a multifaceted defense against environmental challenges.

### Cross-pathway regulation and global effects

The *rel1*-D mutant exhibited alterations in the expression of genes associated with flavonoid biosynthesis, indicating that the genes and metabolites involved in flavonoid metabolism may enhance the biological traits of *rel1*-D. The enhanced production of flavonoids in the *rel1*-D mutant may result from the direct regulation of genes such as *OsCHI*,* OsF3H*,* OsFLS*,* OsCHS*,* OsPAL*, and *Os4CL*, thereby increasing the accumulation of active flavonoids. This research offers new insights into the molecular mechanisms underlying flavonol production in rice.

Moreover, the elevated expression of genes such as *OsHLH*,* OsMPS*, and *OsCDC20.2* suggests that these transcription factors may regulate hormone signaling pathways and cell cycle processes, further enhancing the plant’s adaptive capacity. For instance, OsHLH, a bHLH-class transcription factor, is implicated in rice root development and drought stress response [66]. Given its role in drought tolerance, it is plausible that *OsHLH* also contributes to heat tolerance by modulating the expression of stress-responsive genes. Similarly, OsMPS, an R2R3-type MYB transcription factor, integrates multiple hormone pathways to regulate plant adaptive growth [67]. Its elevated expression in *rel1*-D indicates that it may enhance stress tolerance by fine-tuning hormone signaling and cell wall synthesis-related genes.

This cross-pathway regulation underscores the global effects of *REL1* on plant stress tolerance and highlights its potential for improving rice resilience to high-temperature stress. By integrating metabolic and gene regulatory networks, *REL1* provides a comprehensive defense mechanism that enhances the plant’s ability to withstand multiple stressors. This holistic approach is particularly valuable in the context of climate change, where plants are often subjected to a combination of abiotic stresses.

In summary, the *REL1* mechanism represents a novel and comprehensive approach to enhancing heat tolerance in rice. By integrating metabolic and gene regulatory networks, *REL1* provides a robust defense mechanism against high-temperature stress. This mechanism not only enriches our understanding of rice’s adaptive strategies but also offers valuable insights for breeding programs aimed at developing high-yielding and stress-resistant rice varieties. Future research should focus on further elucidating the regulatory mechanisms of *REL1* and exploring its potential applications in crop improvement. Specifically, studies should investigate the detailed roles of transcription factors such as *OsHLH*,* OsMPS*, and *OsCDC20.2*, as well as the downstream targets of these regulators. Additionally, the potential for translating these findings to other crops should be explored, paving the way for broader applications in agricultural biotechnology.

## Conclusions

Flavonoids constitute a varied category of secondary metabolites that originate from different aglycones and undergo significant chemical alterations, including glycosylation and acylation [68, 69]. In this paper, we summarize the development of flavonoid biosynthesis in rice. Through various approaches, including the analysis of natural mutant metabolomics and transcriptomic studies, we have enhanced our understanding of the structural genes and biochemical pathways involved in flavonoid biosynthesis. The study of T-DNA insertion mutants in rice, complemented by transcriptome sequencing data, provides valuable resources for identifying genes involved in flavonoid biosynthesis.

Exploring the diversity and convergence within the flavonoid biosynthetic pathway not only offers valuable insights into the chemical characteristics of various species but also paves the way for cross-species transformation. Even within a single tissue, flavonoids exhibit remarkable diversity, highlighting the complexity and potential of these compounds. Identifying which of these compounds play a significant role in stress tolerance is a crucial area of research, as it can provide key insights into the adaptive strategies of plants under various environmental pressures. Understanding their functions and relative importance within organisms is particularly challenging, yet essential, given the wide array of flavonoids and their potential roles in stress response. Flavonoids are known to provide a variety of protective benefits for both plants and animals, including antioxidant, anti-inflammatory, and antimicrobial properties. This underscores the urgency and importance of gaining a deeper understanding of the functions of these diverse compounds, as it can have significant implications for both agricultural and medical applications.

## Materials & methods

### Materials and stress treatment of plants

In this study, the wild type used was Oryza sativa ZH11 (WT), while the *rel1*-D mutant was derived from our previous research [36]. All rice plants were cultivated in the paddy fields of Guangzhou, located in Southern China. Metabolomics and transcriptomics sequencing were performed on the tillering stage leaves of both ZH11 and *rel1*-D. For the seedling stage heat treatment, only freshly harvested seeds were used in this study, and they were collected from individual plants to ensure consistency. The dormancy of the seeds was eliminated by drying them in an oven set at 50 °C for five days. A completely randomized design was employed in this study, with six biological replicates for both the control and treatment groups. Each replicate consisted of at least 50 plants to ensure a sufficiently large sample size that could represent the entire population. The plants were randomly assigned to different growth positions to eliminate positional effects. The wild-type plants were planted at the edges of the growth platform to eliminate edge effects. Seedlings at the 3-leaf stage were treated at 42 °C for 48 h. The survival rate of the seedlings was calculated when they were returned to the normal temperature (30 °C) for 0, 2, 4, and 6 days (the percentage of surviving seedlings out of the total seedlings).

### Plant tissue staining

NBT was used to measure the production of superoxide anion in cells. In summary, 50 mg of NBT was added to 100 mL phosphate buffer (pH7.8) to complete dissolution. The NBT solution was obtained. The leaves were immersed in an NBT solution for 6 h. Plant seedlings or leaves were carefully removed with tweezers. The seedlings or leaves were dipped 3 to 5 times in distilled water. Following drying on filter paper, the remaining water was soaked in 95% ethanol for 16 h at 40 °C. The objective of this process was to remove chlorophyll and any light blue background from the plant seedlings or leaves. The procedure involved the repeated replacement of fresh 95% ethanol at various stages.

Cell death was assessed using the TAB method. In summary, leaves were put into test tubes with a trypan blue staining solution (consists of 2.5 mg of trypan blue per milliliter, 25% (wt/v) lactic acid, 23% water-saturated phenol, 25% glycerol, and water), boiled for 10 min and subsequently stored in the dark for 12 h. The leaves were placed in a 25 mg·mL^−1^ chloral hydrate solution for a duration of 24 h to facilitate decolorization. Subsequently, the blue spots present on the leaves were documented and photographed.

In this study, the determination and calculation methods for hydrogen peroxide, lipoxygenase, and proline were carried out according to the instructions of the reagent kits from Nanjing Jiancheng Biotechnology Co., Ltd.

### Transcriptome sequencing

The complete transcriptome sequencing for this research was conducted by Wuhan Metwell Biotechnology Co., Ltd. The cDNA library was sequenced utilizing the Illumina NOVA SEQ 6000 at Gene Denovo Biotechnology in Wuhan, China. The library preparation for sequencing was conducted using the Illumina TruSeq™ RNA sample preparation kit. Total RNA was isolated from the samples utilizing the TRIZOL kit (Invitrogen, Carlsbad, CA) following the guidelines provided by the manufacturer. The RNA purity and concentration were evaluated using an Agilent 2100 bioanalyzer (Agilent Technologies, Palo Alto, CA). To create a single library, a minimum total RNA quantity of 1 µg is required. Additionally, the RNA must have a concentration of at least 35 ng·µL^-1^, an OD 260/280 ratio of 1.8 or greater, and an OD 260/230 ratio of 1.0 or higher. The RNA integrity was confirmed through the use of RNase-free agarose gel electrophoresis.

The Illumina Novaseq 6000 system was engineered specifically for the sequencing of short DNA fragments. The enriched mRNA, which comprises full RNA sequences averaging several kilobases in length, needed to be subjected to random fragmentation. Random fragmentation of mRNA was achieved by introducing a fragmentation buffer, followed by the isolation of small fragments of approximately 300 base pairs through magnetic bead selection. The mRNA enrichment process was carried out using oligomeric (DT) beads from the Epicenter kit in Madison, Wisconsin. The complete mRNA fragments were converted into shorter segments and subsequently reverse transcribed into cDNA utilizing the Illumina UltraRNA library preparation kit (NEB # 7530; New England Biolabs, Ipswich, MA). The purified double-stranded cDNA fragments were then subjected to repair and ligation with the Illumina sequencing linker. The ligated fragments underwent separation through agarose gel electrophoresis combined with PCR. The double-stranded cDNA possesses sticky ends that can be converted into blunt ends by utilizing a terminal repair mixture. An “A” base was then incorporated at the 3’ end to enable the binding of the Y-shaped linker. Ultimately, sequencing was performed utilizing the Illumina platform.

### Extraction, detection and quantitative analysis of metabolites

#### Sample processing

A sample weighing 50 ± 5 mg was introduced into a 2 mL centrifuge tube along with grinding beads measuring 6 mm in diameter. A solution was prepared by combining methanol and water in a 4:1 ratio, which included 0.02 mg·mL-1 of the internal standard L-2-chlorophenylalanine, achieved by adding 400 µL of the mixture. The samples were processed through grinding for a duration of 6 min at a temperature of −10 °C and a frequency of 50 Hz, utilizing a frozen tissue grinder. The procedure entailed a 30-minute ultrasonic extraction performed at a temperature of 5 °C and a frequency of 40 kHz. The samples were preserved at −20 °C for a duration of 30 min before undergoing centrifugation at 13,000 g for 15 min at a temperature of 4 °C. The resulting supernatant was then collected and placed into an injection vial for analysis. A total of 20 µL of supernatant from each sample was collected and pooled to prepare a quality control sample.

#### Quality assurance

Quality Control (QC) samples were prepared by mixing equal volumes of each sample extract. Every QC sample was aligned with the volume of the individual samples and was subjected to the same processing and testing methods as the analytical samples. To ensure the reliability of the data, the relative standard deviation (RSD) of the QC samples was calculated. Features with an RSD greater than 30% were excluded from the analysis to ensure the robustness and repeatability of the method. Additionally, the QC samples were analyzed using principal component analysis (PCA) to assess the consistency and stability of the analytical method. A tight clustering of QC samples in the PCA score plot indicates good method stability and repeatability.

#### LC-MS testing

The examination was performed utilizing the Thermo Fisher UHPLC-Q Exactive system for liquid chromatography-mass spectrometry (LC-MS). The chromatography column employed in this research was the ACQUITY UPLC HSS T3, featuring dimensions of 100 mm × 2.1 mm inner diameter and a particle size of 1.8 μm (Waters, Milford, USA). The mobile phase A consisted of 95% water and 5% acetonitrile, supplemented with 0.1% formic acid. Mobile phase B was composed of 47.5% acetonitrile, 47.5% isopropyl alcohol, and 5% water, with the addition of 0.1% formic acid. The flow rate was set at 0.40 mL·min^-1^, with an injection volume of 5 µL, and the column temperature was maintained at a constant 40 °C. The sample underwent ionization through electrospray ionization, and mass spectrometry signals were recorded in both positive and negative ion scanning modes.

#### Metabolomics methodology and quality control

To ensure the validity of the results, additional quality control steps were implemented. The RSD of the QC samples was calculated for both retention time and peak area to evaluate the separation and analysis method. Typically, an RSD value less than 20% for LC-MS data is considered acceptable. The QC samples were also analyzed using PCA to assess the consistency and stability of the analytical method. A tight clustering of QC samples in the PCA score plot indicates good method stability and repeatability.

#### Verification of RNA-seq data through qRT-PCR

Wu et al. [70] provided a description of the method and calculation formula for qRT − PCR. The primers utilized are listed in Supplementary Table S9.

### Data analysis using statistical methods

The assessment of gene abundance in DGA was performed using RSEM [71], while differential expression analysis was carried out with DESeq2 [72]. A KEGG enrichment analysis was conducted to determine DEGs that are significantly linked to KEGG pathways, employing an adjusted p-value (P_adj) threshold of less than 0.05. Hierarchical Cluster Analysis (HCA) was conducted utilizing online software with the default configurations provided at https://cloud.metware.cn/toolCustom/3. The software available at https://www.omicshare.com/tools/Home/Soft/Become was employed to conduct Principal Component Analysis (PCA) and Orthogonal Partial Least Squares Discriminant Analysis (OPLS-DA). In OriginPro 2021 (OriginLab, Northampton, MA, USA), a bar chart was created. The data obtained from HCA, PCA, and OPLS-DA underwent standardization through Log10 transformation.

## Supplementary Information

Supplementary Material 1.

Supplementary Material 2.

## Data Availability

The datasets supporting the findings of this study are publicly available in the BIG Submission Portal under accession number PRJCA032392 and can be accessed via the following link: https://ngdc.cncb.ac.cn/gsa/browse/CRA020449.
