# Supplementary material for: Integrated transcriptomic and metabolomic analysis unveils heat-tolerance-associated flavonoid metabolites and genes in the rice rel1-D mutant
Source: BMC Genomics. 2025 Sep 1;26:792. doi: 10.1186/s12864-025-11977-0 (PMC12403516; doi:10.1186/s12864-025-11977-0)
Supplement: Supplementary file 1 — Supplementary Material 1. [file 12864_2025_11977_MOESM1_ESM.docx]

**Supplemental Information**


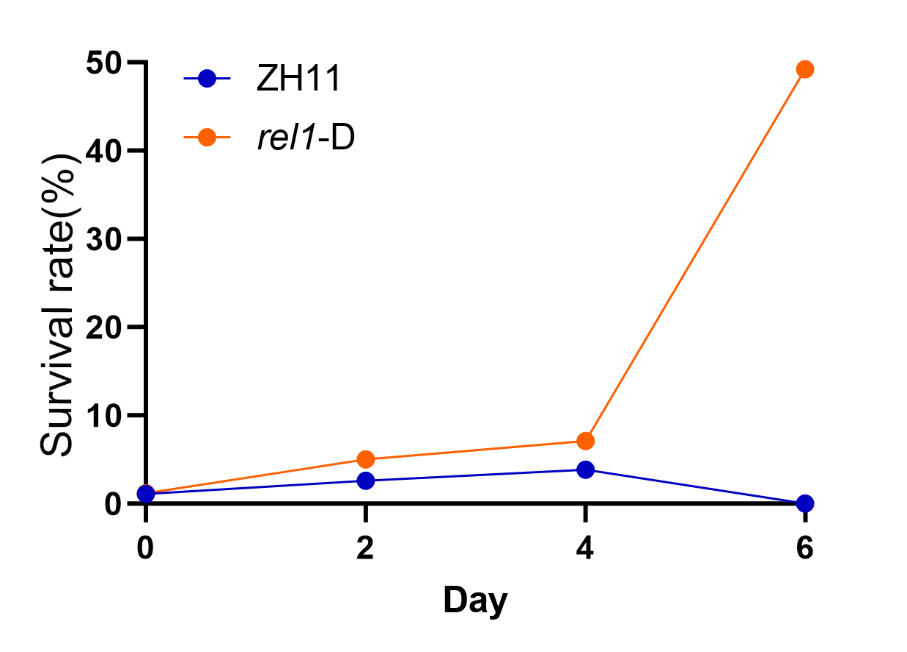


**Fig S1.** Survival rate of rice after high temperature treatment


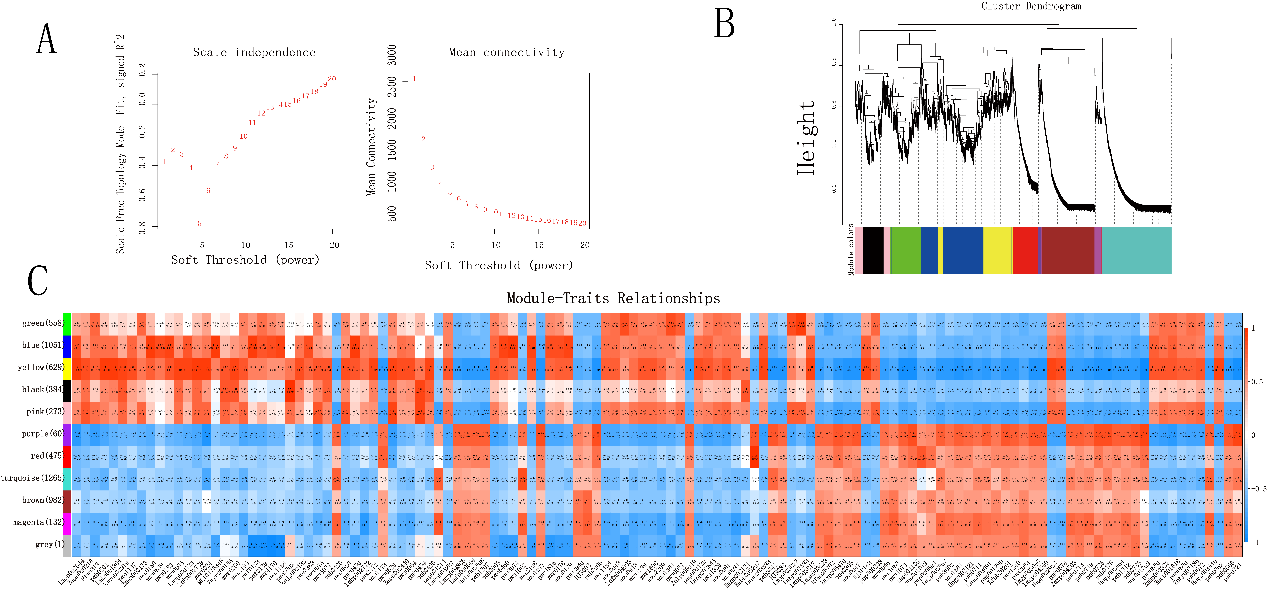


**Fig S2.** WGCNA results obtained using RNA sequencing data. (A)Determination of the adjacency function parameter β in the WGCNA algorithm. (B) Dendrogram showing hierarchical clustering of genes based on dissimilarity of topological overlaps. (C) Correlations between modules and phenotypes.


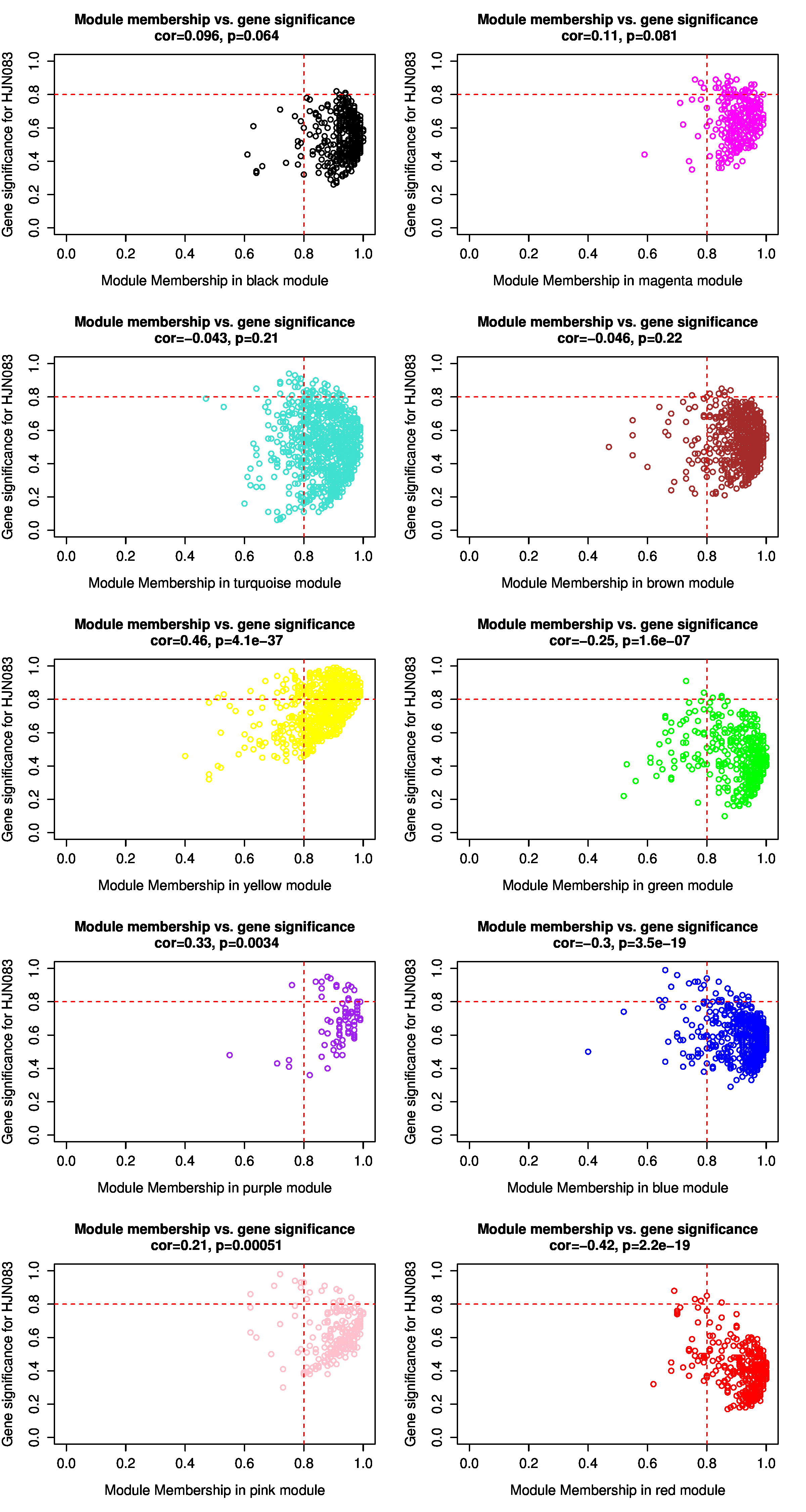


**Fig S3.** GS-MM scatter plot, co-expression network module analysis.
